# A new western Canadian record of *Epeoloides pilosulus* (Cresson), with discussion of ecological associations, distribution and conservation status in Canada

**DOI:** 10.3897/BDJ.6.e22837

**Published:** 2018-04-13

**Authors:** Cory S. Sheffield, Jennifer Heron

**Affiliations:** 1 Royal Saskatchewan Museum, Regina, Canada; 2 British Columbia Ministry of Environment and Climate Change Strategy, Species Conservation Science Unit, Vancouver, Canada

**Keywords:** oil bee, oligolege, Macropis Cuckoo Bee, COSEWIC

## Abstract

**Background:**

*Epeoloides pilosulus*, one of the rarest bees in North America, is a cleptoparasite of *Macropis* bees which themselves are uncommon oligoleges of oil-producing *Lysimachia* flowers. Only two specimens of the cleptoparasite have been reported from Canada since the 1960s, both from Nova Scotia.

**New information:**

A recently collected specimen of *Epeoloides pilosulus* from Alberta, Canada confirms this species from that province and greatly increases its known range in western North America. This record and additional specimens from southern Ontario (one collected in 1978) have implications for the conservation status of this COSEWIC assessed species in Canada, which are discussed.

## Introduction

*Epeoloides pilosulus* (Cresson) (aka Macropis Cuckoo Bee - COSEWIC 2011) is one of the rarest bees in North America. The earliest records in Canada date from at least 1888 (Provancher 1888 and see Sheffield and Perron 2014) and, up to the most recent published accounts of this species in Canada (Sheffield et al. 2004, COSEWIC 2011), there have been only 12 specimens recorded from the country. In the last 60 years, only a handful of specimens have been collected in North America (Sheffield et al. 2004, Wagner and Ascher 2008), with the most recent collection event being a single specimen from Huyck Preserve in Albany County, New York State on July 8, 2014 (http://bugguide.net/node/view/954741) (see Gibbs et al. 2017).

**Figure 1.**
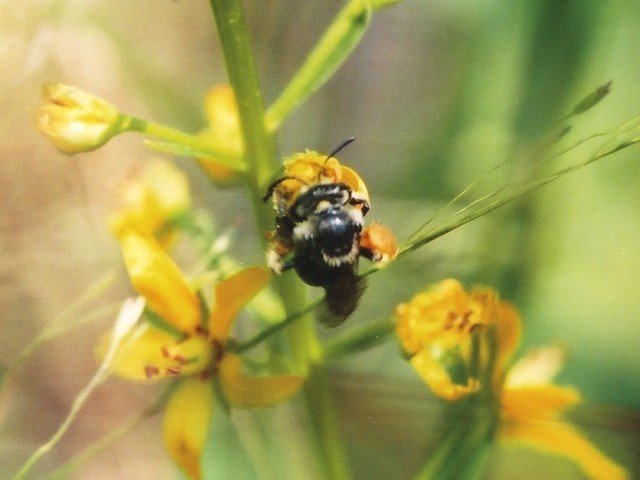
*Macropis nuda* (Melittidae) on oil-producing flowers of *Lysimachia terrestris* (Linnaeus) (Primulaceae) near Middleton, Nova Scotia, Canada. Photo by C.S. Sheffield.

*Epeoloides pilosulus* is a member of one of the most tenuous ecological existences seen in North American plant-pollinator relationships, being a cuckoo (=cleptoparasite) of and thus, dependent on *Macropis* bees (Melittidae) which in turn are uncommon oligoleges of oil-producing *Lysimachia* species (Primulaceae, Myrsinoideae) (Sheffield et al. 2004, Wagner and Ascher 2008, Alves-dos-Santos 2009) (Fig. 1).

*Epeoloides pilosulus* is one of only two globally recognised species in the genus (Michener 2007), the other, *E. coecutiens* (Fabricius), which was studied in detail by Straka and Bogusch (2007b) is found in Europe (Popov 1958, Pekkarinen et al. 2003, Bogusch 2005, Michez and Patiny 2005, Dötterl and Schäffler 2006). Interestingly, these two species are presently considered members of the tribe Osiriini, a taxon otherwise restricted to the Neotropics (Michener 2007), though others have indicated that *Epeoloides* may have affiliations to other tribes in the Apidae (Straka and Bogusch 2007a, Cardinal et al. 2010). However, the known hosts of *Epeoloides* (i.e. *Macropis* bees) and their host plants (genus *Lysimachia*) have centres of diversity elsewhere (Michez and Patiny 2005, Michener 2007). In fact, both *Macropis* (Michez and Patiny 2005) and *Lysimachia* (Chen and Hu 1979, Zhang et al. 2006, Zhou et al. 2015) are most diverse in China; eight of the 16 *Macropis* species and 138 of the ca. 180 known plant species occurring there (Michez and Patiny 2005, Zhang et al. 2006), though *Lysimachia* also occurs in areas where neither genus of bee has been recorded (Marr and Bohm 1997). However, not all species of *Lysimachia* are oil-producing and, of the five recognised subgenera, only two contain species with oil-producing flowers that are co-occuring with *Macropis* bees (Michez and Patiny 2005). Michez and Patiny (2005) provide a detailed account of the biogeography of *Macropis* bees and *Lysimachia* hosts. Strangely, *Epeoloides* is seemingly absent from the centres of diversity of both *Macropis* bees and *Lysimachia* floral hosts (Michener 2007).

The purpose of this paper is to report on a recently collected specimen of *E. pilosulus* from Alberta, Canada which significantly increases the range of this species in western North America and additional specimens in the collections at the Royal Saskatchewan Museum and the University of Guelph. We also comment on its conservation status in Canada.

## Materials and methods

Data from *Epeoloides pilosulus* specimens known from Canada were previously compiled for the conservation assessment for the Committee On the Status of Endangered Wildlife in Canada (COSEWIC) in 2011 (COSEWIC 2011). Data from that report were combined with previously unrecorded Canadian records not available or known at that time, including material collected in a Malaise trap as part of efforts to collect material within Canada's National Parks for DNA barcoding, a single specimen from Saskatchewan within the entomology collection at the Royal Saskatchewan Museum and two specimens (collected in 1915 and 1978) in the entomology collection at the University of Guelph.

## Data resources

The full dataset for *Epeoloides* and *Macropis* specimens that were used in this study is archived with Canadensys (http://community.canadensys.net/) under resource title “A new western Canadian record of Epeoloides pilosulus (Cresson)” and can be accessed using the following: doi: https://doi.org/10.5886/vfi8nn.

## Taxon treatments

### Epeoloides pilosulus

(Cresson, 1878)

Nomada pilosula Cresson 1878: 77 [♂]
Nomia compacta Provancher 1888: 337 [♂] [synonymy by Mitchell (1962)]
Epeolus pilosulus Provancher 1888: 426 [♀] [synonymy by Mitchell (1962)]
Viereckella obscura Swenk 1907: 299 [♀] [synonymy by C.D. Michener, as cited in Sheffield et al. (2004) and see Michener (2000)]
Viereckella ceanothina Cockerell, in Swenk 1907: 300 [♂] [synonymy of *V. obscura* by Swenk (1912)]
Epeoloides nearcticus Ducke 1909: 39 [♀] [synonymy by Bequaert (1920)]

#### Materials

- **Type status:** Other material. **Occurrence:** catalogNumber: CNC_Epeoloides_pilosulus_1; recordedBy: A.R. Brooks; individualCount: 1; sex: female; lifeStage: adult; **Taxon:** scientificName: Epeoloides pilosulus; kingdom: Animalia; phylum: Arthropoda; class: Insecta; order: Hymenoptera; family: Apidae; genus: Epeoloides; specificEpithet: pilosulus; scientificNameAuthorship: (Cresson, 1878); vernacularName: Macropis Cuckoo Bee; **Location:** continent: North America; country: Canada; stateProvince: Saskatchewan; locality: Wood Mountain; decimalLatitude: 49.316; decimalLongitude: -106.38; **Identification:** identifiedBy: T. Romankova; dateIdentified: 2001; **Event:** year: 1955; month: 8; day: 5; **Record Level:** collectionCode: Insects; ownerInstitutionCode: CNC; basisOfRecord: PreservedSpecimen
- **Type status:** Other material. **Occurrence:** catalogNumber: CNC_Epeoloides_pilosulus_2; recordedBy: F.W.L. Sladen; individualCount: 1; lifeStage: adult; **Taxon:** scientificName: Epeoloides pilosulus; kingdom: Animalia; phylum: Arthropoda; class: Insecta; order: Hymenoptera; family: Apidae; genus: Epeoloides; specificEpithet: pilosulus; scientificNameAuthorship: (Cresson, 1878); vernacularName: Macropis Cuckoo Bee; **Location:** continent: North America; country: Canada; stateProvince: Manitoba; locality: Aweme; decimalLatitude: 49.7; decimalLongitude: -99.6; **Identification:** identifiedBy: T. Romankova; dateIdentified: 2001; **Event:** year: 1919; month: 7; day: 13; **Record Level:** collectionCode: Insects; ownerInstitutionCode: CNC; basisOfRecord: PreservedSpecimen
- **Type status:** Other material. **Occurrence:** catalogNumber: CNC_Epeoloides_pilosulus_3; recordedBy: S.M. Clark; individualCount: 1; lifeStage: adult; **Taxon:** scientificName: Epeoloides pilosulus; kingdom: Animalia; phylum: Arthropoda; class: Insecta; order: Hymenoptera; family: Apidae; genus: Epeoloides; specificEpithet: pilosulus; scientificNameAuthorship: (Cresson, 1878); vernacularName: Macropis Cuckoo Bee; **Location:** continent: North America; country: Canada; stateProvince: Ontario; locality: One Sided Lake; decimalLatitude: 49.0602778; decimalLongitude: -93.8947223; **Identification:** identifiedBy: T. Griswold; **Event:** year: 1960; month: 7; day: 24; **Record Level:** collectionCode: Insects; ownerInstitutionCode: CNC; basisOfRecord: PreservedSpecimen
- **Type status:** Other material. **Occurrence:** catalogNumber: CNC_Epeoloides_pilosulus_4; recordedBy: F.W.L. Sladen; individualCount: 1; lifeStage: adult; **Taxon:** scientificName: Epeoloides pilosulus; kingdom: Animalia; phylum: Arthropoda; class: Insecta; order: Hymenoptera; family: Apidae; genus: Epeoloides; specificEpithet: pilosulus; scientificNameAuthorship: (Cresson, 1878); vernacularName: Macropis Cuckoo Bee; **Location:** continent: North America; country: Canada; stateProvince: Québec; locality: Aylmer; decimalLatitude: 45.39; decimalLongitude: -75.84; **Identification:** identifiedBy: H.L. Viereck; **Event:** year: 1915; month: 6; day: 21; **Record Level:** collectionCode: Insects; ownerInstitutionCode: CNC; basisOfRecord: PreservedSpecimen
- **Type status:** Other material. **Occurrence:** catalogNumber: JBWM0296405; recordedBy: R.D. Bird; individualCount: 1; lifeStage: adult; **Taxon:** scientificName: Epeoloides pilosulus; kingdom: Animalia; phylum: Arthropoda; class: Insecta; order: Hymenoptera; family: Apidae; genus: Epeoloides; specificEpithet: pilosulus; scientificNameAuthorship: (Cresson, 1878); vernacularName: Macropis Cuckoo Bee; **Location:** continent: North America; country: Canada; stateProvince: Manitoba; locality: Aweme; decimalLatitude: 49.7; decimalLongitude: -99.6; **Identification:** identifiedBy: H.L. Viereck; **Event:** year: 1924; month: 7; day: 6; **Record Level:** collectionCode: Insects; ownerInstitutionCode: Wallis-Roughley Museum of Entomology; basisOfRecord: PreservedSpecimen
- **Type status:** Other material. **Occurrence:** catalogNumber: 632961; recordedBy: J.D. Ritchie; individualCount: 1; lifeStage: adult; **Taxon:** scientificName: Epeoloides pilosulus; kingdom: Animalia; phylum: Arthropoda; class: Insecta; order: Hymenoptera; family: Apidae; genus: Epeoloides; specificEpithet: pilosulus; scientificNameAuthorship: (Cresson, 1878); vernacularName: Macropis Cuckoo Bee; **Location:** continent: North America; country: Canada; stateProvince: Saskatchewan; locality: Wallwort; decimalLatitude: 52.5; decimalLongitude: -104.04; **Identification:** identifiedBy: C.D. Michener; **Event:** year: 1942; month: 7; day: 16; **Record Level:** collectionCode: Insects; ownerInstitutionCode: Natural History Museum and Biodiversity Research Center, University of Kansas; basisOfRecord: PreservedSpecimen
- **Type status:** Other material. **Occurrence:** catalogNumber: 632964; recordedBy: J.D. Ritchie; individualCount: 1; lifeStage: adult; **Taxon:** scientificName: Epeoloides pilosulus; kingdom: Animalia; phylum: Arthropoda; class: Insecta; order: Hymenoptera; family: Apidae; genus: Epeoloides; specificEpithet: pilosulus; scientificNameAuthorship: (Cresson, 1878); vernacularName: Macropis Cuckoo Bee; **Location:** continent: North America; country: Canada; stateProvince: Saskatchewan; locality: Wallwort; decimalLatitude: 52.5; decimalLongitude: -104.04; **Identification:** identifiedBy: C.D. Michener; **Event:** year: 1942; month: 7; day: 17; **Record Level:** collectionCode: Insects; ownerInstitutionCode: Natural History Museum and Biodiversity Research Center, University of Kansas; basisOfRecord: PreservedSpecimen
- **Type status:** Other material. **Occurrence:** catalogNumber: 632958; recordedBy: J.D. Ritchie; individualCount: 1; lifeStage: adult; **Taxon:** scientificName: Epeoloides pilosulus; kingdom: Animalia; phylum: Arthropoda; class: Insecta; order: Hymenoptera; family: Apidae; genus: Epeoloides; specificEpithet: pilosulus; scientificNameAuthorship: (Cresson, 1878); vernacularName: Macropis Cuckoo Bee; **Location:** continent: North America; country: Canada; stateProvince: Saskatchewan; locality: Wallwort; decimalLatitude: 52.5; decimalLongitude: -104.04; **Identification:** identifiedBy: C.D. Michener; **Event:** year: 1942; month: 7; day: 20; **Record Level:** collectionCode: Insects; ownerInstitutionCode: Natural History Museum and Biodiversity Research Center, University of Kansas; basisOfRecord: PreservedSpecimen
- **Type status:** Other material. **Occurrence:** catalogNumber: BBHYK681-10; recordedBy: BIObus 2010; individualCount: 1; lifeStage: adult; **Taxon:** scientificName: Epeoloides pilosulus; kingdom: Animalia; phylum: Arthropoda; class: Insecta; order: Hymenoptera; family: Apidae; genus: Epeoloides; specificEpithet: pilosulus; scientificNameAuthorship: (Cresson, 1878); vernacularName: Macropis Cuckoo Bee; **Location:** continent: North America; country: Canada; stateProvince: Alberta; locality: Elk Island National Park, Hayburger Trail; decimalLatitude: 53.634; decimalLongitude: -112.857; **Identification:** identifiedBy: J.K. Stahlhut; **Event:** year: 2010; month: 8; day: 11; **Record Level:** collectionCode: Insects; ownerInstitutionCode: University of Guelph, Centre for Biodiversity Genomics; basisOfRecord: PreservedSpecimen
- **Type status:** Other material. **Occurrence:** catalogNumber: BEECB232-07; recordedBy: C.S. Sheffield; individualCount: 1; sex: male; lifeStage: adult; **Taxon:** scientificName: Epeoloides pilosulus; kingdom: Animalia; phylum: Arthropoda; class: Insecta; order: Hymenoptera; family: Apidae; genus: Epeoloides; specificEpithet: pilosulus; scientificNameAuthorship: (Cresson, 1878); vernacularName: Macropis Cuckoo Bee; **Location:** continent: North America; country: Canada; stateProvince: Nova Scotia; locality: near Middleton; decimalLatitude: 44.9665; decimalLongitude: -65.056; **Identification:** identifiedBy: C.S. Sheffield; dateIdentified: 2003; **Event:** year: 2002; month: 7; day: 17; **Record Level:** collectionCode: Insects; ownerInstitutionCode: University of Guelph, Centre for Biodiversity Genomics; basisOfRecord: PreservedSpecimen
- **Type status:** Other material. **Occurrence:** catalogNumber: 632963; recordedBy: C.S. Sheffield; individualCount: 1; sex: male; lifeStage: adult; **Taxon:** scientificName: Epeoloides pilosulus; kingdom: Animalia; phylum: Arthropoda; class: Insecta; order: Hymenoptera; family: Apidae; genus: Epeoloides; specificEpithet: pilosulus; scientificNameAuthorship: (Cresson, 1878); vernacularName: Macropis Cuckoo Bee; **Location:** continent: North America; country: Canada; stateProvince: Nova Scotia; locality: near Middleton; decimalLatitude: 44.9665; decimalLongitude: -65.056; **Identification:** identifiedBy: C.S. Sheffield; dateIdentified: 2003; **Event:** year: 2002; month: 7; day: 11; **Record Level:** collectionCode: Insects; ownerInstitutionCode: Natural History Museum and Biodiversity Research Center, University of Kansas; basisOfRecord: PreservedSpecimen
- **Type status:** Lectotype. **Occurrence:** catalogNumber: 1529 [yellow label]; recordedBy: L. Provancher; individualCount: 1; sex: female; lifeStage: adult; **Taxon:** scientificName: Epeoloides pilosulus; originalNameUsage: Epeolus pilosulus Provancher, 1888; kingdom: Animalia; phylum: Arthropoda; class: Insecta; order: Hymenoptera; family: Apidae; genus: Epeoloides; specificEpithet: pilosulus; scientificNameAuthorship: (Cresson, 1878); vernacularName: Macropis Cuckoo Bee; **Location:** continent: North America; country: Canada; stateProvince: Québec; locality: Cap Rouge; decimalLatitude: 46.76; decimalLongitude: -71.35; **Identification:** identifiedBy: L. Provancher; **Record Level:** collectionCode: Insects; ownerInstitutionCode: The Provancher Collection, Université Laval, Ste Foy, Québec; basisOfRecord: PreservedSpecimen
- **Type status:** Holotype. **Occurrence:** catalogNumber: 1670 [yellow label]; recordedBy: L. Provancher; individualCount: 1; sex: male; lifeStage: adult; **Taxon:** scientificName: Epeoloides pilosulus; originalNameUsage: Nomia compacta Provancher, 1888; kingdom: Animalia; phylum: Arthropoda; class: Insecta; order: Hymenoptera; family: Apidae; genus: Epeoloides; specificEpithet: pilosulus; scientificNameAuthorship: (Cresson, 1878); vernacularName: Macropis Cuckoo Bee; **Location:** continent: North America; country: Canada; stateProvince: Québec; locality: Cap Rouge; decimalLatitude: 46.76; decimalLongitude: -71.35; **Identification:** identifiedBy: L. Provancher; **Record Level:** collectionCode: Insects; ownerInstitutionCode: The Provancher Collection, Université Laval, Ste Foy, Québec; basisOfRecord: PreservedSpecimen
- **Type status:** Other material. **Occurrence:** catalogNumber: RSKM_ENT_E-81549; recordedBy: J.D. Ritchie; individualCount: 1; lifeStage: adult; **Taxon:** scientificName: Epeoloides pilosulus; kingdom: Animalia; phylum: Arthropoda; class: Insecta; order: Hymenoptera; family: Apidae; genus: Epeoloides; specificEpithet: pilosulus; scientificNameAuthorship: (Cresson, 1878); vernacularName: Macropis Cuckoo Bee; **Location:** continent: North America; country: Canada; stateProvince: Saskatchewan; locality: Wallwort; decimalLatitude: 52.5; decimalLongitude: -104.04; **Identification:** identifiedBy: C.S. Sheffield; dateIdentified: 2016; **Event:** year: 1942; month: 7; day: 10; **Record Level:** collectionCode: Insects; ownerInstitutionCode: RSKM; basisOfRecord: PreservedSpecimen
- **Type status:** Other material. **Occurrence:** catalogNumber: debu01088907; recordedBy: G.J. Spencer; individualCount: 1; sex: male; lifeStage: adult; **Taxon:** scientificName: Epeoloides pilosulus; kingdom: Animalia; phylum: Arthropoda; class: Insecta; order: Hymenoptera; family: Apidae; genus: Epeoloides; specificEpithet: pilosulus; scientificNameAuthorship: (Cresson, 1878); vernacularName: Macropis Cuckoo Bee; **Location:** continent: North America; country: Canada; stateProvince: Ontario; locality: Simcoe; decimalLatitude: 42.83; decimalLongitude: -80.3; **Identification:** identifiedBy: R. Lambert; dateIdentified: 1952; **Event:** year: 1915; month: 7; day: 2; **Record Level:** collectionCode: Insects; ownerInstitutionCode: DEBU; basisOfRecord: PreservedSpecimen
- **Type status:** Other material. **Occurrence:** catalogNumber: debu01088908; recordedBy: D. Morris; individualCount: 1; sex: female; lifeStage: adult; **Taxon:** scientificName: Epeoloides pilosulus; kingdom: Animalia; phylum: Arthropoda; class: Insecta; order: Hymenoptera; family: Apidae; genus: Epeoloides; specificEpithet: pilosulus; scientificNameAuthorship: (Cresson, 1878); vernacularName: Macropis Cuckoo Bee; **Location:** continent: North America; country: Canada; stateProvince: Ontario; locality: Milton; decimalLatitude: 43.51; decimalLongitude: -79.88; **Identification:** identifiedBy: T. Romankova; **Event:** year: 1978; month: 7; day: 6; **Record Level:** collectionCode: Insects; ownerInstitutionCode: DEBU; basisOfRecord: PreservedSpecimen

#### Conservation

The species is assessed nationally as Endangered in Canada by COSEWIC (COSEWIC 2011), but has not been listed under the Canadian Species At Risk Act (SARA). The criteria COSEWIC uses to assess the status of species is adapted from the International Union for the Conservation of Nature (IUCN) Red-list categories (see COSEWIC 2012). *Epeoloides pilosulus* was assessed under the B criteria (i.e. meeting B2ab(iii) as the index of area of occupancy (4 km², calculated only for the last ten year period of assessment) was below the threshold of 500 km², the species had been found at only one location in the past 10 years, has always existed in fragmented populations and there is a continuing decline in suitable wetland habitat for the flowering plant species upon which the host cuckoo bee ultimately depends due to development, invasive species and reduction in wetland area) (COSEWIC 2011).

Each province and territory has separate legislation that protects species at risk in that jurisdiction. Though there are previous records of *Epeoloides pilosulus* from Nova Scotia, Quebec, Ontario, Manitoba and Saskatchewan, the species is only listed as a Species at Risk under the Nova Scotia Endangered Species Act (NSESA 2013). The species was assessed as Data Deficient by the Committee on the Status of Species at Risk in Ontario (COSSARO) (COSSARO 2010).

Non-legal conservation status ranks have also been completed using NatureServe methodology and definitons (Master et al. 2012). The global rank is G1 (critically imperilled - because of extreme rarity [often 5 or fewer occurrences] or because of some factor(s) such as very steep declines making it especially vulnerable to extirpation from the state/province). The national rank for (Canada) is N1 (critically inmperilled), with provincial ranks for Nova Scotia (S1), Quebec (SNR, unranked - conservation status not yet assessed), Ontario (S1), Manitoba (S1) and Saskatchwan (S1) (CESCC 2016, Atlantic Conservation Data Centre 2017).

The four recent records from Alberta in 2010, Ontario in 1978 (both reported here) and Nova Scotia in 2002 (Sheffield et al. 2004) are the only specimens of this species collected in Canada since 1960.

## Analysis

**Figure 2.**
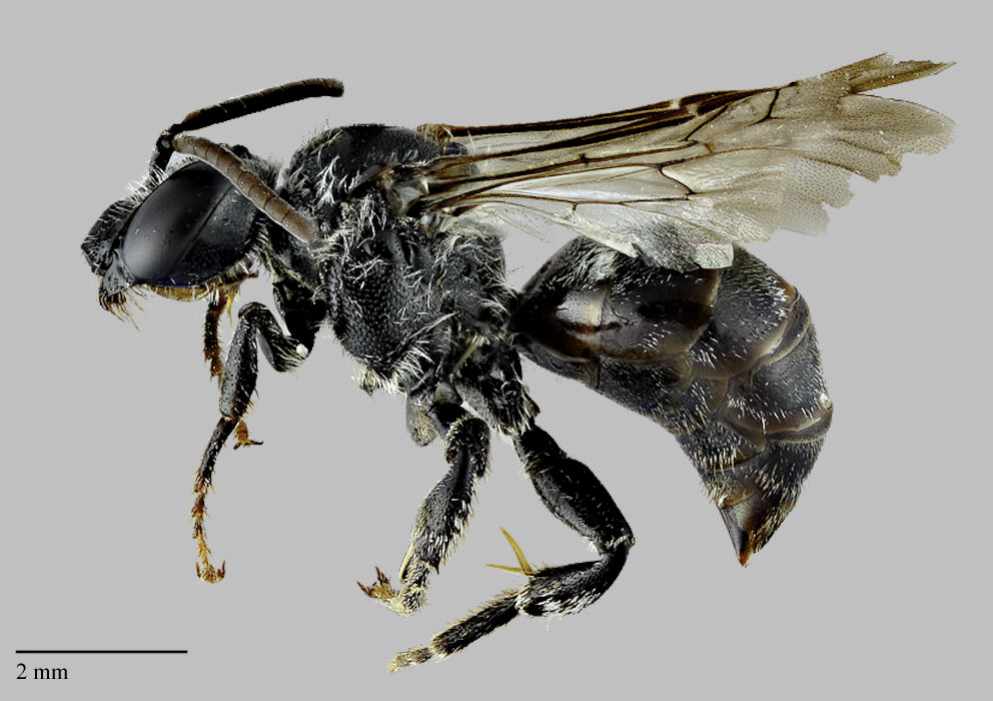
The Macropis Cuckoo Bee, *Epeoloides pilosulus* (Cresson) (Apidae). Female, lateral view. Specimen from Alberta. Photo by R. Oram, Royal Saskatchewan Museum.

**Figure 3.**
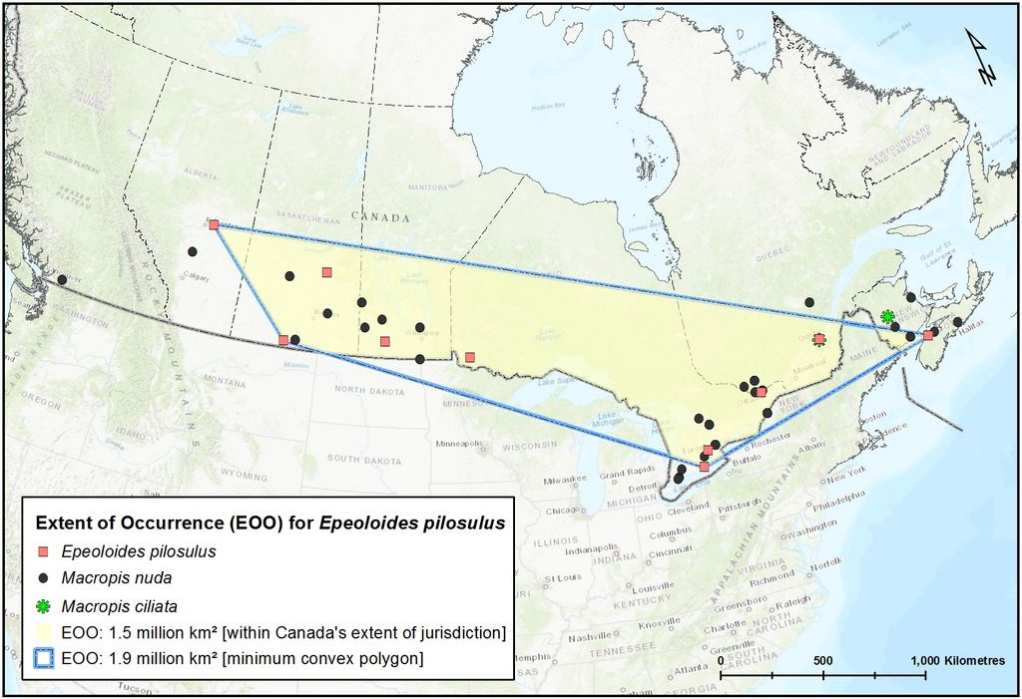
The known Extent of Occurrence (EOO) of *Epeoloides pilosulus* (Cresson) (Apidae) within Canada's extent of jurisdiction (yellow area) and based on minimum convex polygon (within blue lines).

**Figure 4.**
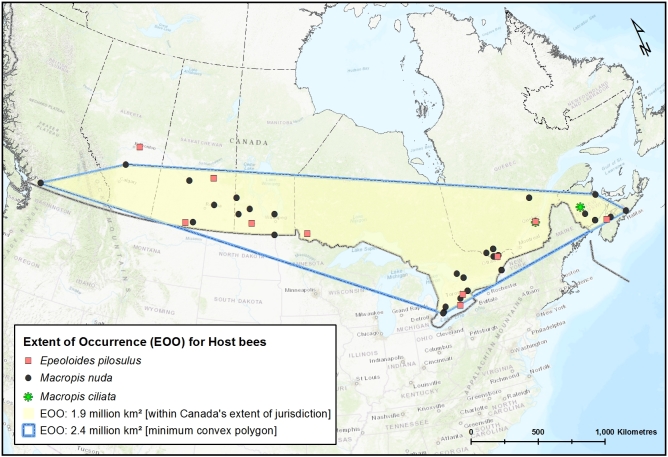
The known Extent of Occurrence (EOO) of *Macropis* bees, *M. nuda* (Provancher), *M. ciliata* Patton (Melittidae) within Canada's extent of jurisdiction (yellow area) and based on minimum convex polygon (within blue lines). Data from the Canadian National Collection of Insects, Arachnids and Nematodes (CNC), the Royal Saskatchewan Museum (RSKM) and other sources (Suppl. material 1).

**Figure 5.**
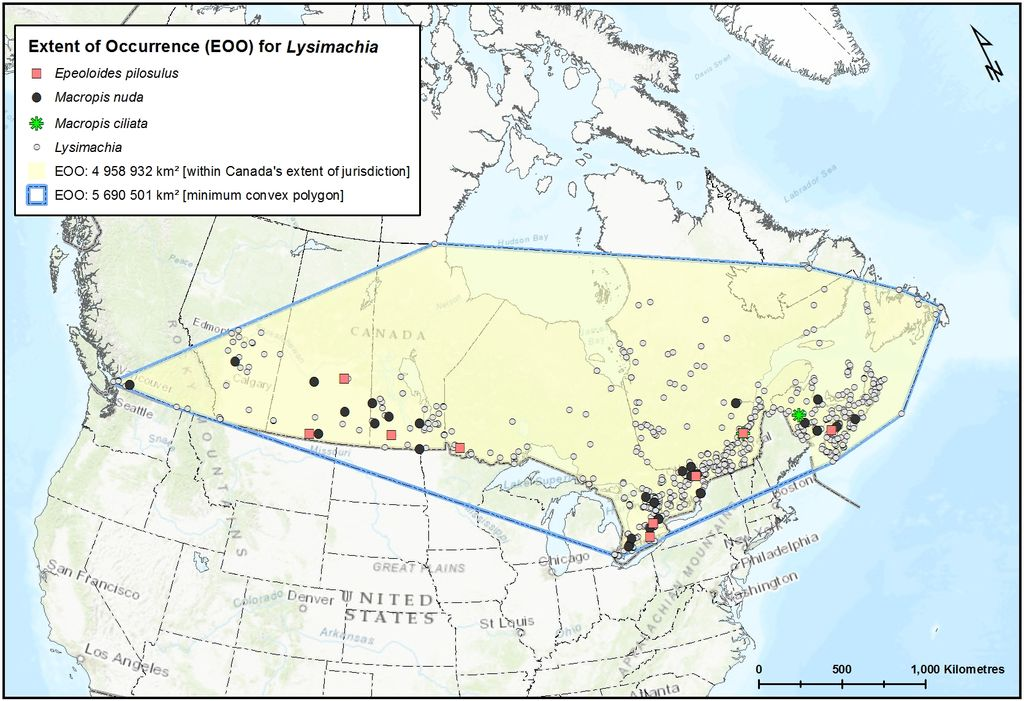
Extent of Occurrance (EOO) of native oil-producing *Lysimachia* species (Primulaceae) in Canada. Figure produced using records obtained from GBIF for species listed in Table 1 (see GBIF 2018, and Suppl. material 2).

Since the time of the COSEWIC assessment of this species for Canada (COSEWIC 2011), the new records from Alberta (Elk Island National Park) (Fig. 2) and southern Ontario increases the Extent of Occurrence (EOO) in Canada from ca. 0.91 million km^2^ (204,490 km^2^ was reported by COSEWIC 2011) to ca. 1.5 million km^2^ and the Area of Occupancy (AO, based on 2 x 2 = 4 km^2^ grids) from 24 km^2^ to 160 km^2^ (Fig. 3). As this species is dependent on and confined within the range of its host bees (*Macropis*) (Fig. 4; EOO of 1.9 million km^2^ in Canada) which in turn are dependent on oil-producing species of *Lysmiachia*, the range of suitable native host plants in Canada represents the possible range extent of the cuckoo bee, which is almost 5 million km^2^ (Fig. 5); likely it is even greater considering that at least some species of *Lysimachia* occur in the Northwest Territories and Yukon (Porsild and Cody 1980, Cody 2000). However, *E. pilosulus* has not previously been recorded this far west in North America (Ascher and Pickering 2018); the genus was not even included in the key to bee genera of north-western North America (Stephen et al. 1969).

## Discussion

**Figure 7.**
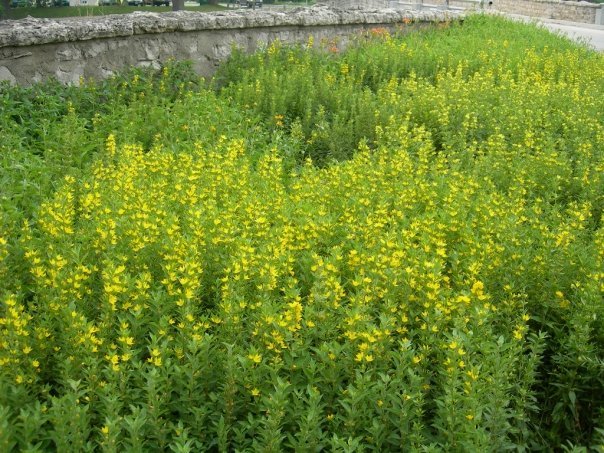
A non-native oil producing species, *Lysimachia punctata* L. (Primulaceae), growing as an ornamental in Guelph, Ontario. Photo by C. Sheffield.

The newly recorded Canadian specimen of *E. pilosulus* greatly increases the recorded EOO of this species in Canada (Fig. 3) and for North America, being the furthest into the northwest that this species has been observed. Recently, but not suprisingly due to the presence of the host plant(s), the main *Macropis* host in Canada, *M. nuda* (Provancher), was photographed in south-central British Columbia (see Kline 2017), though specimens have been known from that province since 1914 (Fig. 4), suggesting that *E. pilosulus* may also be present in that province. The genus *Lysimachia* however is more widespread than either bee species in this triumvariate, with two species, *L. thyrsiflora* and *L. ciliata* occuring well into the north of Canada and Alaska (Table 1). Porsild and Cody (1980) recorded *L. thyrsiflora* from the Northwest Territories, but this species is a non-oil producer, so is likely not being used by *Macropis* bees as a source of oil. The other northernly distributed species, *L. ciliata* is an oil-producer (Fig. 6) and is used by *Macropis* bees in southern parts of the plants range, though neither this genus nor the cuckoo bee have been recorded that far north, suggesting that the bees, but not the plant, are dependants within these plant-pollinator (+ cuckoo) relationships. However, there are also six non-native *Lysimachia* species in North America, four of which are oil-producers (Table 1) and most of these are grown ornamentally (Fig. 7).

Assessing the conservation status of cuckoo bees is challenging, particularly because these species are dependent on the presence of their host(s), are typically present in low abundance due to their specialised life histories and, therefore, are not as commonly collected as non-parasitic bees (Sheffield et al. 2013b). Additionally, the host(s) of very few cuckoo bees are known (Michener 2007, Sheffield et al. 2013b), so the threats to host(s) may be unknown. Most of the recently collected specimens of *E. pilosulus* have been captured through passive methods of sampling; specimens collected in 2002 in Nova Scotia (Sheffield et al. 2004) and 2005-2006 in Connecticut (Wagner and Ascher 2008, Wagner et al. 2014) were from pan traps. While these traps are easily set, choosing the appropriate habitat can be challenging because little is known about the microhabitat characteristics of the cuckoo bee in North America, though somewhat known for the host (see Rozen and Jacobson 1980, Cane et al. 1983). *Macropis* bees are relatively widespread but uncommon, though potential host plants are also widely distributed (Fig. 5). Presence and abundance of *Lysimachia* are not strong indicators of the host bee’s presence (COSEWIC 2011) and both are not uniformly distributed throughout the landscape. Therefore, it is also assumed these factors also limit the population of *E. pilosulus*.

In the past decade, interest in bee conservation has grown and sampling for bees has increased substantially, though much of this has been project specific and new records have been identified as part of larger studies (Sheffield et al. 2013a, Wagner et al. 2014), as opposed to efforts specifically targeting this species throughout its range (COSEWIC 2011). However comprehensive sampling for bees continues to be challenging and some cuckoo bee species can be rare within certain landscapes, have a limited flight period synchronised with the host(s) and/or may have population fluctuations, making their detection difficult (Sheffield et al. 2013b). However, for *E. pilosulus* and other cuckoos of oil bees (Alves-dos-Santos 2009), it may be possible to develop specific sampling methods to accurately assess the bee’s presence in the landscape based on chemical ecology. For example, European species of *Macropis* and *E. coecutiens* (and likely its North American counter) find the flowers and nests of its *Macropis* hosts, respectively by the scent of floral oils (Dötterl and Schäffler 2006, Dotterl 2008), so developing a chemical lure for *E. pilosulus* in North American as a tool for conservation monitoring may be possible.

## Supplementary Material

Supplementary material 1 Specimens of Macropis bees from Canada used for map generation Data type: occurences
Brief description: File contains occurence data for *Macropis* bee (Hymenoptera: Melittidae) specimens from Canada.
File: oo_194268.xls Cory S. Sheffield, Jennifer Heron

Supplementary material 2 Occurence data of Lysimachia species occurring in Canada and the United States. Data type: occurence
Brief description: Download Information:
DOI: https://doi.org/10.15468/dl.4mglnz (may take some hours before being active)
Creation Date: Friday, 30 March 2018 22:42:31 o'clock GMT
Records included: 20723 records from 112 published datasets
Data size: 5.2 MB
Download format: DWCA
Filter used: Country: Canada or United States
TaxonKey: Lysimachia L.
File: oo_194287.txt GBIF.org

XML Treatment for Epeoloides pilosulus

## Figures and Tables

**Figure 6a.**
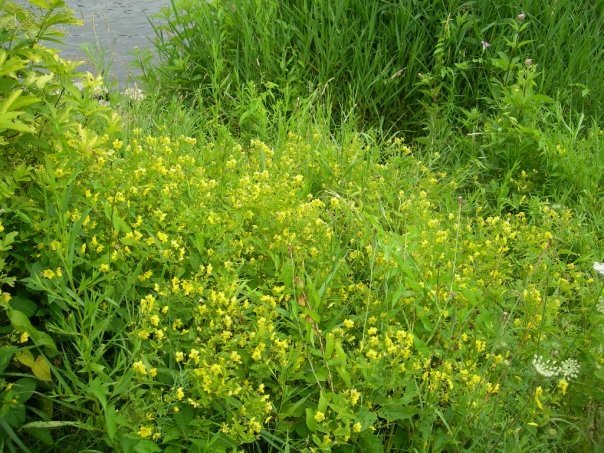
Growing at river's edge, Speed River, Guelph Ontario. Photo by C. Sheffield.

**Figure 6b.**
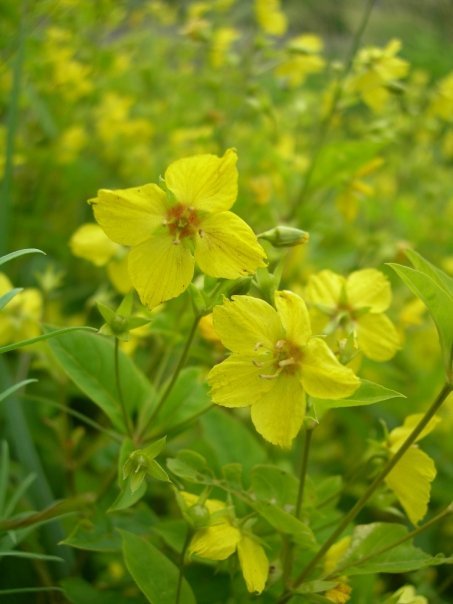
Flowers up close. The central reddish areas are oil-producing glands.

**Table 1.** Species of *Lysmiachia* in North America, showing their native status, oil production and notes on distribution. Compiled from data in Cody (2000), Coffey and Jones (1980), Estes et al. (2015), Porsild and Cody (1980), Ray (1956), USDA (2017).

***Lysimachia* Species**	**Subgenus**	**Native**	**Oil**	**Distribution**
*L. asperulifolia* Poir.	* Lysimachia *	yes	yes	Southeast USA
*L. barystachys* Bunge	* Palladia *	no	no	Southeast USA
*L. ciliata* L.	* Seleucia *	yes	yes	CAN, USA [incl. AK]
*L. clethroides* Duby	* Palladia *	no	no	Eastern CAN and USA
L. × commixta Fernald [*terrestris*× thyrsiflora]	* Lysimachia *	yes	yes	Eastern CAN and USA
*L. fraseri* Duby	* Lysimachia *	yes	yes	Southeast USA
*L. graminea* (Greene) Hand.-Maz.	* Seleucia *	yes	yes	Southeast USA
*L. hybrida* Michx.	* Seleucia *	yes	yes	CAN, USA
*L. japonica* Thunb.	* Lysimachia *	no	yes	Southeast USA
*L. lanceolata* Walter	* Seleucia *	yes	yes	Eastern CAN and USA
*L. lewisii* Estes, Shaw & Mausert-Mooney	* Seleucia *	yes	yes	Southeast USA
*L. loomisii* Torr.	* Lysimachia *	yes	yes	Southeast USA
*L. nummularia* L.	* Nummularia *	no	yes	CAN, USA
L. × producta (A. Gray) Fernald (pro sp.) [*quadrifolia*× terrestris]	* Lysimachia *	yes	yes	Eastern CAN and USA
*L. punctata* L.	* Lysimachia *	no	yes	CAN, USA
*L. quadriflora* Sims	* Seleucia *	yes	yes	Central CAN and USA
L. × radfordii H.E. Ahles [*loomisii*× quadrifolia]	* Lysimachia *	yes	yes	Southeast USA
*L. radicans* Hook.	* Seleucia *	yes	yes	Southern USA
*L. terrestris* (L.) Britton, Sterns & Poggenb.	* Lysimachia *	yes	yes	CAN, USA [not AK, YT, NT]
*L. thyrsiflora* L.	* Naumburgia *	yes	no	CAN, USA [including AK, YT, NT]
*L. tonsa* (Alph. Wood) Alph. Wood ex Pax & R. Knuth	* Seleucia *	yes	yes	Southeast USA
*L. vulgaris* L.	* Lysimachia *	no	yes	CAN, USA
